# Visualized trends and bibliometric analysis in ankle cartilage repair from 2004 to 2024

**DOI:** 10.3389/fmed.2024.1503707

**Published:** 2024-11-20

**Authors:** Xuefei Fu, Zhixing Zhang, Yingxiang Wang, Lin Lu, Tao Chen, Haobin Deng, Hao Li, Defu Yu

**Affiliations:** ^1^Department of Orthopedics, Anhui No.2 Provincial People’s Hospital, Hefei, China; ^2^School of Medicine, Nankai University, Tianjin, China; ^3^Beijing Key Laboratory of Regenerative Medicine in Orthopedics, Key Laboratory of Musculoskeletal Trauma and War Injuries PLA, Institute of Orthopedics, Chinese PLA General Hospital, Beijing, China; ^4^Department of Radiotherapy, Anhui No.2 Provincial People’s Hospital, Hefei, China; ^5^Department of Oncology, Liuzhou People’s Hospital Affiliated to Guangxi Medical University, Liuzhou, China; ^6^School of Clinical Medicine, Anhui Medical College, Hefei, China

**Keywords:** bibliometric, citation, ankle cartilage injury, repair, hotspots

## Abstract

Ankle cartilage injuries are a common sports-related condition that significantly impairs patients’ daily activities and imposes substantial economic burdens on both families and society. Effective cartilage repair strategies are crucial to addressing this pathological condition. Current conservative treatments include muscle strengthening, use of ankle braces, physical therapy, and the administration of NSAIDs. In cases of severe injury, surgical interventions such as osteophyte resection and cartilage transplantation may be necessary. However, the inherent regenerative capacity of articular cartilage is limited, and conventional treatments are insufficient to promote cartilage regeneration and repair. Consequently, innovative therapies such as stem cell therapy, exosome therapy, and cartilage regeneration scaffolds are prioritized for future development. In recent years, significant progress has been made in ankle cartilage repair. While bibliometric studies on cartilage repair exist, specific analyses focused on ankle cartilage repair are lacking. This study aims to address this gap by conducting a bibliometric analysis of 131 articles published over the past two decades, highlighting development trajectories, research hotspots, and evolutionary trends through knowledge mapping. Our findings indicate growing global interest, with the United States leading in international collaboration, funding, publication output, and citation frequency. *Foot & Ankle International* emerges as the leading journal for publication and dissemination in this field, with Kerkhoffs GMMJ identified as the most influential author. Notable hotspot keywords include “osteochondral lesions” and “platelet-rich plasma.” By highlighting critical research hotspots and collaboration patterns, this study not only enriches the existing literature on ankle cartilage repair but also serves as a foundational resource for clinicians and researchers aiming to develop innovative strategies for improving patient outcomes. Furthermore, our findings underscore the necessity of interdisciplinary collaboration in advancing the understanding and treatment of ankle cartilage injuries. Ultimately, the visual characterization of these trends provides valuable insights into the field’s evolutionary trajectory, offering guidelines for future research directions and encouraging further exploration of this promising area.

## 1 Introduction

The ankle joint, the most weight-bearing joint in the human body, endures forces of 5 to 7 times body weight during standing (1). It comprises the tibia, fibula, and talus, all covered by a thin layer of cartilage with an average thickness of approximately 1.6 millimeters on the joint surface (2). The incidence of ankle sprains ranges from 19.0 to 26.6 per 1,000 person-years, with around 2 million cases annually in the United States (3). Approximately half of these cases involve damage to the ankle cartilage, impacting both the joint surface and subchondral bone (4). Ankle cartilage injuries are predominantly associated with traumatic events, particularly in high-impact sports such as football (5). The primary demographic affected includes males aged 20 to 30 years, who are typically more physically active (6). With the growing number of fitness enthusiasts, the prevalence of ankle cartilage injuries is likely to increase, highlighting the urgent need for effective prevention and treatment strategies. Indeed, Ankle cartilage injury can indeed progress into osteoarthritis (OA), a complex condition influenced by both biomechanical factors and inflammation. Several studies emphasize the role of biomechanics and inflammation in the onset and progression of ankle cartilage injuries, underscoring their importance in understanding how these injuries can lead to OA (7, 8). The ankle, highly prone to ligament injuries, often faces joint instability after cartilage damage, which may further contribute to OA development. Additionally, ankle proprioception is recognized as a key factor influencing joint stability, which is particularly relevant in individuals with knee OA. Research has also explored the link between ankle cartilage injury and subsequent OA development. For instance, Song et al. (9) reviewed the mechanistic connections between lateral ankle sprains, chronic ankle instability (CAI), and post-traumatic osteoarthritis (PTOA), highlighting the cascade of events following ligamentous trauma and its impact on OA risk (9). Understanding the interactions between ankle cartilage injury, joint instability, and OA development is essential to reducing the health burden of OA, and future research should prioritize identifying effective treatments to prevent and manage OA in patients with ankle injuries.

The primary characteristic of ankle cartilage injury is chronic ankle pain, often accompanied by localized discomfort within the ankle joint induced by compression (10). This pain is frequently associated with intermittent edema, restricted range of motion, and exacerbated swelling and pain with increased mobility (11). Clinically, the diagnosis is typically established through MRI and CT scans. Articular cartilage is a highly specialized tissue that lacks undifferentiated cells necessary for repair and does not possess blood vessels to provide nutritional support, significantly limiting its intrinsic capacity for self-repair (12). In clinical practice, conventional treatments focus on strengthening the surrounding muscles, such as through weight-bearing calf raises, and restricting excessive movement with the use of ankle bandages. Physical therapy techniques, including ultrashort wave, infrared ray, and shock wave therapies, are also commonly applied. Additionally, medications like non-steroidal anti-inflammatory drugs (NSAIDs), glucosamine sulfate supplements, intra-articular sodium hyaluronate, and glucocorticoids are frequently administered (13, 14). However, these traditional therapies generally offer limited efficacy, and prolonged use of NSAIDs and glucocorticoids can lead to severe side effects, such as gastrointestinal ulcers and perforations (15).

The exploration of surgical methods to repair ankle cartilage is ongoing, with common techniques including reparative and replacement strategies. Reparative techniques, such as bone marrow stimulation (BMS) performed under arthroscopy, involve penetrating the subchondral bone to allow mesenchymal stem cells to migrate into the lesion area, where they can form fibrocartilage and repair small defects (16). Replacement techniques, on the other hand, include autologous or allogeneic chondrocyte transplantation and osteochondral grafting, which are typically employed for larger lesions (17). In addition to these, biomimetic scaffolds that possess superior mechanical properties and biocompatibility have emerged as promising alternatives to natural grafts. Some of these scaffolds are designed to incorporate drugs, cytokines, and stem cells to enhance the repair process (18). While these surgical treatments have demonstrated promising short- and medium-term outcomes, there remains a lack of sufficient long-term evidence, and as a result, no definitive consensus or guidelines have been established (4).

In response to the limitations of traditional and surgical therapies, advanced cartilage repair techniques, such as concentrated bone marrow aspirate (cBMA) therapy and platelet-rich plasma (PRP) therapy, have been developed to optimize the biological environment and improve treatment outcomes (19). cBMA can be harvested from various sources within the human body, such as iliac bone marrow, utilizing mesenchymal and hematopoietic stem cells for therapeutic purposes (20). PRP therapy enhances the biological milieu by promoting mesenchymal stem cell migration to the injury site, combating inflammation, and reducing pain through the improvement of synovial fluid quality (21). Cell-free polymer-based scaffold implantation has been explored as a treatment option for cartilage defects, with clinical studies discussing various advantages and disadvantages of different strategies. For instance, a randomized controlled trial demonstrated that cell-free scaffold resulted in a significant improvement in the American Orthopedic Foot and Ankle Society (AOFAS) hindfoot score compared to traditional microfracture (22). Finite element analysis has been utilized to assess the impact of talar osteochondral defects on treatment prognosis, emphasizing the need for accurate prediction of injury depth (23). Furthermore, T1ρ relaxation mapping has been utilized to assess osteochondral lesions of the talus, with researchers conducting analysis and interpretation of experimental data related to hyaline cartilage at the ankle joint (24). Furthermore, tissue engineering presents a promising avenue for addressing ankle cartilage injuries by enabling the regeneration of functional cartilage through various advanced techniques. One approach involves cultivating tissue constructs using chondrogenic cells, scaffolds, and bioreactors to support tissue development and maturation (25). Additionally, the integration of materials, biological factors, gene therapy, and cell therapy in biphasic composite scaffolds shows promise in regenerating osteochondral defects (26). Novel scaffold designs, such as gelatin-calcium-phosphate biphasic scaffolds, have been utilized in double-chamber bioreactors to engineer cartilage constructs successfully (27). Mesenchymal stem cells (MSCs) also offer a robust therapeutic approach due to their self-renewal capability, immunomodulatory properties, and potential for differentiation into multiple lineages (28). For example, synovium-derived stem cells have been identified as potential candidates for cartilage regeneration due to their chondrogenic potential and minimal hypertrophic differentiation (29). Furthermore, MSC-derived exosomes have emerged as innovative biomarkers and therapeutic agents for treating ankle cartilage injuries. These exosomes can be engineered to enhance their biological activity and targeting capabilities, as well as to facilitate large-scale production (30). Surface-modified and drug-loaded exosomes offer the potential for cell-free therapies, representing a promising advancement in tissue engineering (31). Emerging techniques like 3D bioprinting with *in situ* crosslinking have addressed the limitations of traditional scaffold designs, allowing for the creation of bioinks with desirable printability, cytocompatibility, and bioactivity tailored for cartilage tissue repair (32). In summary, tissue engineering offers a comprehensive approach for ankle cartilage repair by integrating advances in cell and gene therapy, scaffold design, and bioreactor technology.

Before embarking on further basic and clinical investigations, it is essential to comprehensively summarize the current research focus and frontiers related to ankle cartilage injury. Bibliometrics, which utilizes quantitative analysis techniques such as mathematical and statistical methods, provides objective scientific indicators that enable researchers to track quantitative changes, distributions, and patterns within the existing literature (33). This approach offers critical data and a comprehensive view of dynamic trends, assisting researchers in evaluating current challenges, identifying key institutions, and assessing the quantity and quality of regional publications (34). Moreover, bibliometrics plays a pivotal role in forecasting potential future research and development directions. The insights derived from bibliometric analysis can be particularly valuable for government policymakers, guiding decisions related to funding allocations and other strategic areas (35, 36). As a result, bibliometrics is widely recognized and utilized as a vital tool in research evaluations. Given its many advantages, it is unsurprising that bibliometrics continues to gain traction among scholars and researchers worldwide.

Despite the growing interest in ankle cartilage regeneration and repair within the scientific community, there is a noticeable lack of bibliometric investigations that examine the evolution and analytical appraisal of this research field. This article aims to address this gap by assessing the global publication patterns of articles related to ankle cartilage regeneration and repair. To achieve this objective, we have systematically organized and evaluated data on the distribution of publications, stratified by country, author, journal, and impact. Additionally, we have analyzed the frequency and timing of keywords to present trends through bibliometric maps and predict potential future developments in this field. By providing a comprehensive analysis of the global development patterns in ankle cartilage regeneration and repair, this study seeks to enhance readers’ understanding and serve as a valuable contemporary resource for prospective collaborative endeavors and clinical applications.

## 2 Materials and methods

### 2.1 Literature sources and research methods

In recent years, bibliometric analysis has been widely utilized to examine specific topics, fields of influence and practice, knowledge bases, and emerging hotspots. It offers unique advantages over traditional review articles, meta-analyses, and experimental studies. Citation networks, for instance, can summarize publication trends, predict research hotspots, and provide deeper insights into the frontiers of specific fields. Currently, popular bibliometric software includes CiteSpace, VOSviewer, UCINET, Scimat, Pajek, and Bicomb. For this study, publications related to ankle cartilage injury research were sourced from the Science Citation Index Expanded (SCI-Expanded) database within Clarivate Analytics’ Web of Science Core Collection (WoSCC). Following established methodologies from previous studies, relevant research on ankle cartilage injury was identified and subjected to bibliometric and visualization analyses. The search parameters were set for publications dated between May 1, 2004, and May 1, 2024, using the following search formula: Theme = “ankle cartilage” OR “ankle chondral” OR “ankle osteochondral” OR “talus cartilage” OR “talar cartilage” OR “talar chondral” OR “talar osteochondral” OR “distal tibia cartilage” OR “distal tibia chondral” OR “distal tibia osteochondral” OR “tibiotalar cartilage” OR “tibiotalar chondral.” The inclusion criteria for publications on “osteochondral” injuries were as follows: Primary methods employed in treating ankle cartilage injuries. Limited to articles and reviews as the literature types. Papers must be written in English.

**Figure 1:**
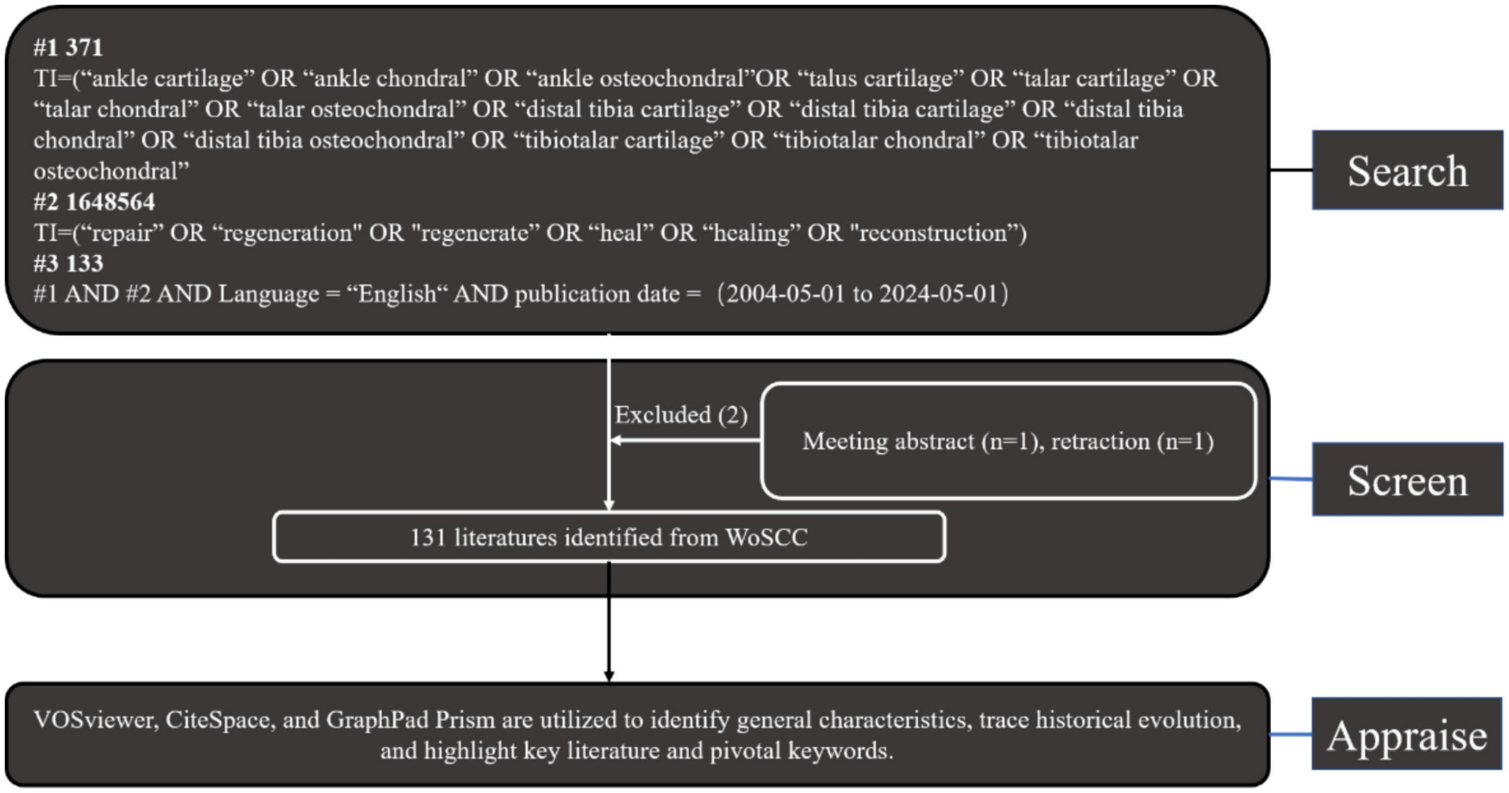
Flowchart illustrating the literature selection process.

Exclusion criteria included meeting abstracts and retractions. Two reviewers (XFF and ZXZ) meticulously evaluated these publications, manually filtering out any deemed irrelevant to the research topic of ankle cartilage injury. Additionally, experienced corresponding authors were consulted for adjudication on the inclusion of potentially relevant publications that were initially excluded (Figure 1). Finally, all documents were imported into CiteSpace, VOSviewer, and R bibliometrix for separate visualization analyses.

### 2.2 Data acquisition and search strategies

To begin, the annual trend of publications and relative research interest (RRI) over the years were analyzed and visualized using the curve-fitting function of GraphPad Prism 8. The world map was generated using R software, incorporating python with numpy, scipy, and matplotlib libraries (37). The time curve of publications was plotted following the methodology outlined in a previous article (37). Next, VOSviewer (version 1.6.17) software was employed to construct and visualize: the collaboration analysis among countries/regions and institutions. The co-citation analysis of journals, authors, and references. The co-occurrence analysis of keywords. Following this, CiteSpace (version 6.3.R1), developed by Professor Shen et al. (38), was utilized to construct and visualize: A dual-map overlay for journals. Cluster analysis of co-cited keywords and references. The detection of authors, references, and keywords with intense citation bursts. The CiteSpace parameters were configured as follows: Time span: 2004–2024; Years per slice: 1; Link retaining factor (LRF): 3; Look back years (LBY): 5; E for top N: 1; Link strength: cosine; Link scope: within slices; Selection criteria: g-index with *k* = 25. These settings were chosen to optimize the analysis and provide a comprehensive visualization of the data.

## 3 Results

### 3.1 Analysis of global literature publication trend

**Figure 2:**
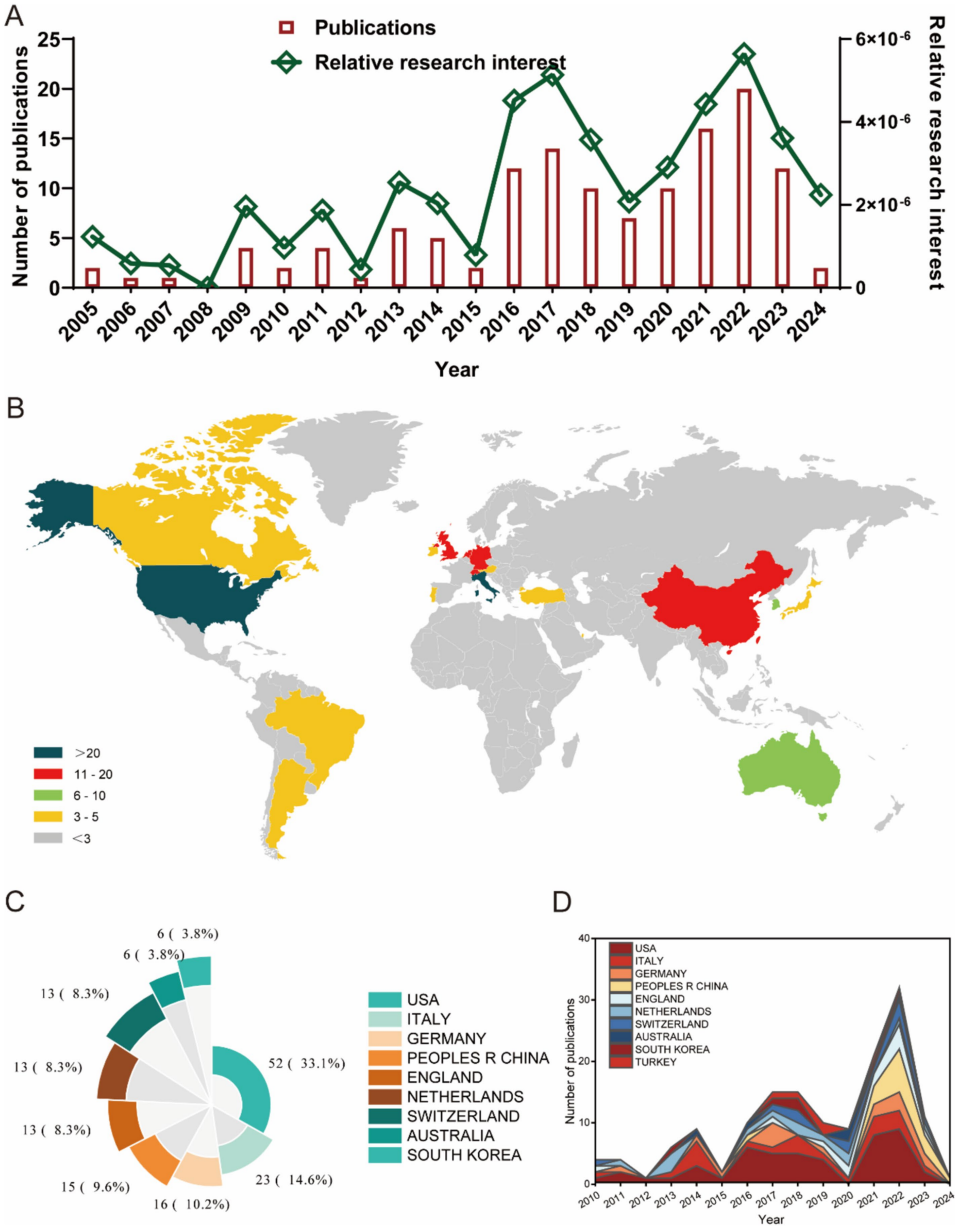
Global trends and countries/regions contributing to the research field regarding ankle cartilage repair from 2004 to 2024. (A) The global number (red bars) of publications and relative research interests (green curve) related to ankle cartilage repair from 2004 to 2024. (B) Distribution of ankle cartilage repair research in world map. (C) The sum of publications related to ankle cartilage repair from the top 9 countries/regions. (D) The annual number of publications in the top 10 most productive countries from 2004 to 2024.

We summarized the publication and distribution trends of global literature on ankle cartilage repair (Figure 2). As illustrated in Figure 2A, from 2004 to 2015, the annual number of publications in this field seldom exceeded five articles. However, starting in 2016, there was a significant upward trend in annual publications, peaking at 20 articles in 2022. The relative research interest (RRI) in ankle cartilage repair mirrored this trend, suggesting that the field is increasingly becoming a focal point of research. The apparent decline in 2024 for both indicators is likely due to the timing of data collection, as 2024 is not yet complete and many articles remain unpublished and, therefore, were not included in the statistics.

Figures 2B,C show that a total of 65 countries/regions have published English-language literature on ankle cartilage repair. The United States leads with the highest number of publications (52 articles, 33.1%), followed by Italy (23 articles, 14.6%), Germany (16 articles, 10.2%), China (15 articles, 9.6%), the United Kingdom (13 articles, 8.3%), the Netherlands (13 articles, 8.3%), Switzerland (13 articles, 8.3%), Australia (6 articles, 3.8%), and South Korea (6 articles, 3.8%). The annual number of publications from the top 10 contributing countries/regions (Figure 2D) increased from 10 in 2016 to 33 in 2022. Notably, China began publishing articles in this field only in 2021, yet its contribution has been substantial within this short period.

Overall, global research on ankle cartilage repair has experienced rapid development, particularly since 2016, drawing increasing attention from the scientific community.

### 3.2 Citation analysis of global literature

**Figure 3:**
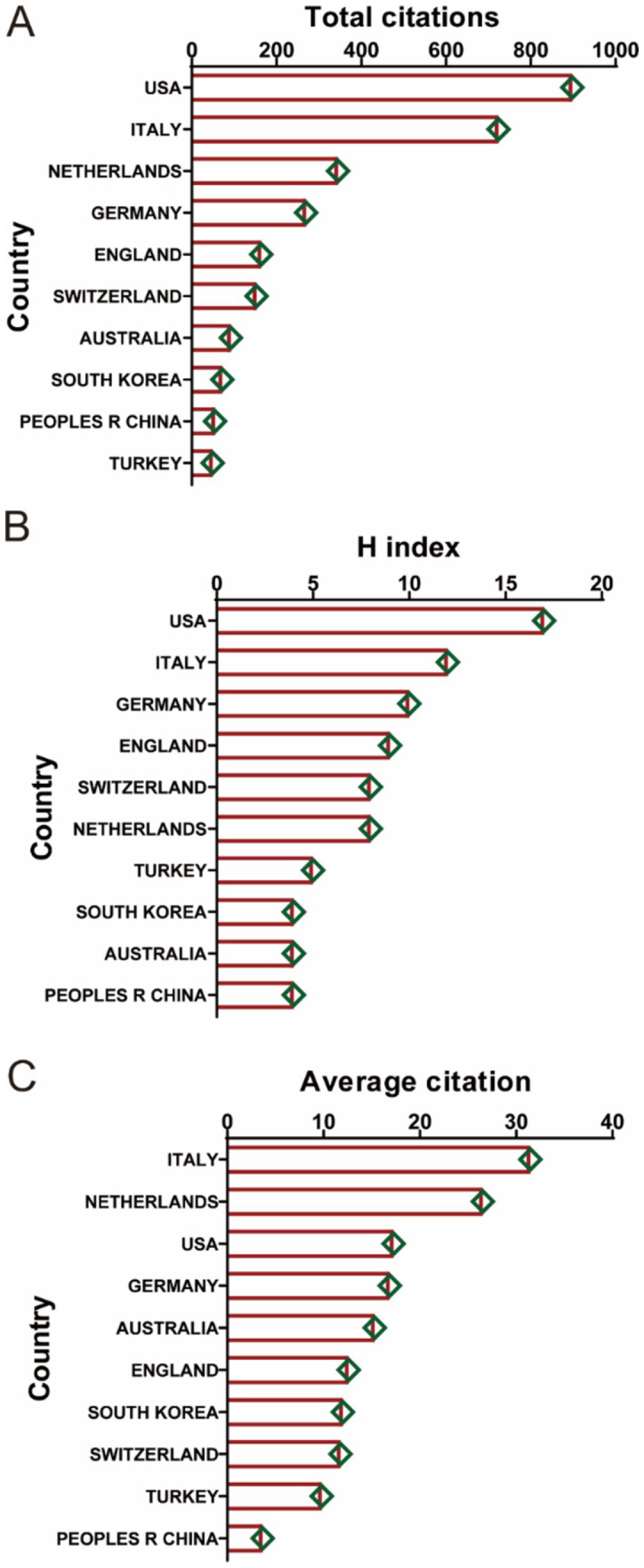
(A) The top 10 countries/regions of total citations regarding ankle cartilage repair from 2004 to 2024. (B) The top 10 countries/regions of the publication H-index related to ankle cartilage repair from 2004 to 2024. (C) The top 10 countries/regions of the average citations per publication related to ankle cartilage repair from 2004 to 2024.

Figure 3 illustrates the citation metrics for the top 10 countries/regions in the field of ankle cartilage repair. In terms of total citations (Figure 3A), the United States leads with the highest citation frequency (900 citations), followed by Italy with 700 citations, both significantly ahead of other countries. The Netherlands and Germany also have notable citation counts, though specific figures for these countries need to be clarified based on the data. Regarding the H-index, which measures both the productivity and citation impact of the publications, the United States and Italy again rank highest, indicating a strong influence in the field. Germany and the United Kingdom follow in the H-index rankings. Italy and the Netherlands exhibit the highest average citation frequency per publication, indicating that, on average, articles from these countries are cited more often than those from other countries. The United States ranks third in terms of average citation frequency, reflecting both its high volume of publications and their substantial impact in the field.

### 3.3 Analysis of national/regional and institutional cooperation in global literature

**Figure 4:**
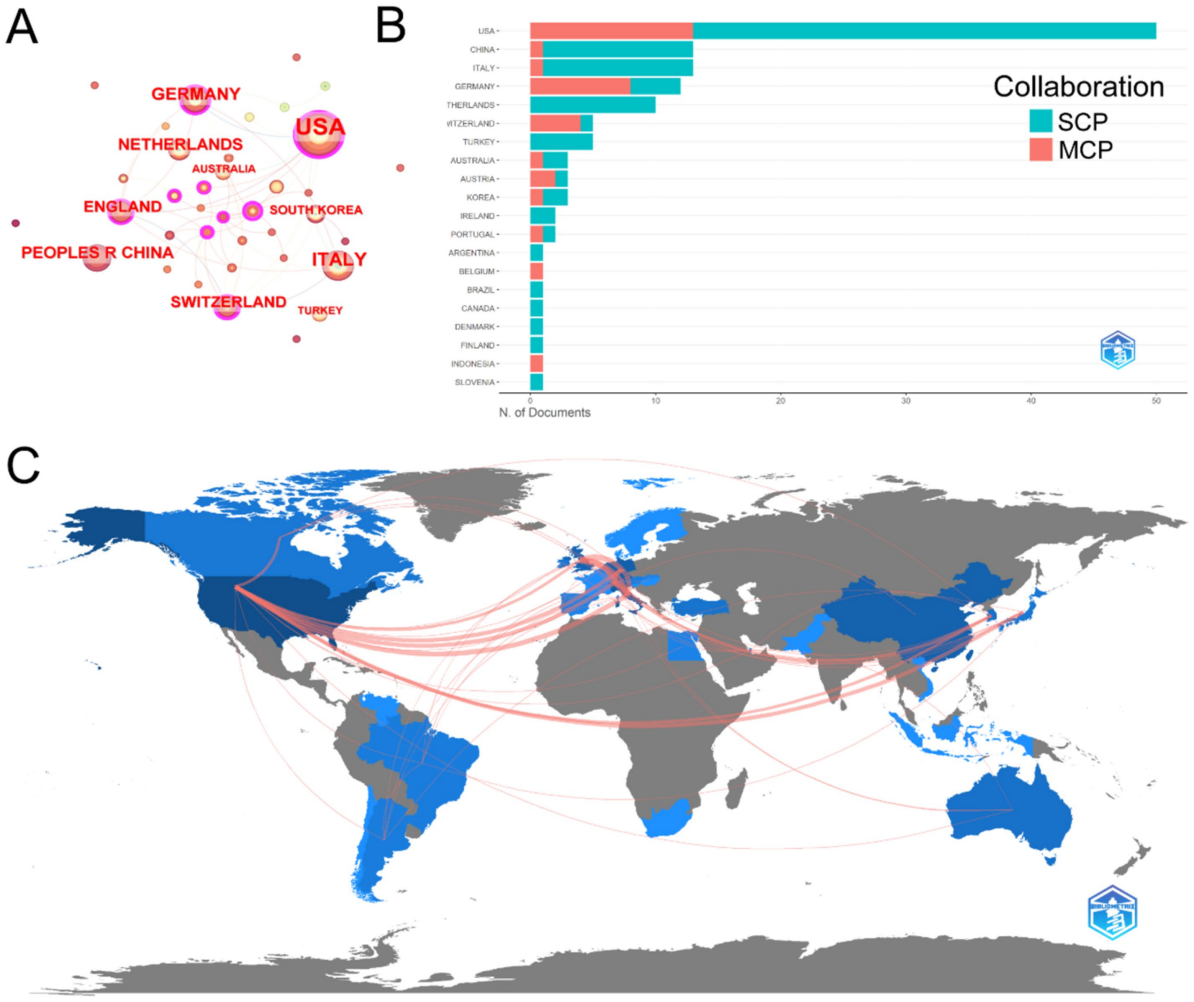
(A) Country/regional collaboration analysis. (B) Type of collaboration of the top 20 most productive countries/regions. (C) The geographical network map of country/regional collaboration in ankle cartilage repair.

Figure 4A provides a detailed analysis of country/region collaboration in the field of ankle cartilage repair. In Figure 4A, the concentric circles represent countries/regions, with the color and thickness of the rings indicating the year and number of publications, respectively. The connecting lines represent collaborative relationships, with the thickness of the lines reflecting the degree of collaboration between countries. Nodes highlighted in purple denote countries with high centrality in the collaboration network, indicating that the United States, Germany, Switzerland, the United Kingdom, and South Korea play pivotal roles in international collaboration.

Figure 4B categorizes the collaboration types of publications from the 20 most productive countries. SCP (single country publications) refers to articles authored by researchers from the same country, while MCP (multiple country publications) refers to articles authored by researchers from different countries. The United States, with the highest number of publications, leads in both SCP and MCP. Germany and Switzerland exhibit extensive international collaborations, ranking second and third in MCP. In terms of SCP, Italy and China rank second and third, respectively.

Figure 4C presents a geographical network map that visually displays the publication and collaboration landscape in the field of ankle cartilage repair. The United States and Western Europe emerge as the primary regions conducting research in this area, with strong collaborative ties among them. East Asia, South America, and Australia also contribute to the field, though collaboration with China appears to be less extensive.

Table 1 lists the top 10 institutions contributing to research in ankle cartilage repair. Italy’s Istituto Ortopedico Rizzoli (IRCCS) and the Netherlands’ University of Amsterdam are tied for first place, each with 11 articles. They are followed by the United States’ Hospital for Special Surgery, which has published 9 articles. Among these top institutions, 5 are from the United States, 3 from Italy, and 2 from the Netherlands, highlighting the leading role of research institutions from these three countries, particularly the United States, in this field (Table 2). Table 3 enumerates the top 10 major funding sources for research related to ankle cartilage repair. The list includes 4 funding sources from the United States, 2 from Germany, and one each from China, the Netherlands, Austria, and Switzerland. This distribution underscores the substantial investment from the United States in advancing research in ankle cartilage repair.

**Table 1:** The top 10 institutions published literature related to ankle cartilage repair from 2004 to 2024.

Rank	Institution	Article counts	Percentage (%)
1	Irccs Istituto Ortopedico Rizzoli	11	8.397
2	University of Amsterdam	11	8.397
3	Hosp Special Surg	9	6.87
4	Academic Medical Center Amsterdam	8	6.107
5	University of Bologna	7	5.344
6	Duke University	5	3.817
7	Irccs Istituto Ortopedico Galeazzi	5	3.817
8	Pennsylvania Commonwealth System of Higher Education Pcshe	5	3.817
9	Rush University	5	3.817
10	University of California System	5	3.817

**Table 2:** The top 10 well-represented research areas.

Rank	Research Areas	Records	Percentage (%)
1	Orthopedics	100	76.336
2	Surgery	32	24.427
3	Sport Sciences	24	18.321
4	Rheumatology	9	6.87
5	Radiology Nuclear Medicine Medical Imaging	8	6.107
6	Engineering	7	5.344
7	Cell Biology	3	2.29
8	General Internal Medicine	2	1.527
9	Biotechnology Applied Microbiology	2	1.527
10	Biophysics	2	1.527

**Table 3:** The top 10 funds related to ankle cartilage repair from 2004 to 2024.

Rank	Funds	Article counts	Percentage
1	National Institutes of Health Nih USA	6	4.58
2	United States Department of Health Human Services	6	4.58
3	National Natural Science Foundation of China Nsfc	3	2.29
4	Nih National Institute of Arthritis Musculoskeletal Skin Diseases Niams	3	2.29
5	Amsterdam Umc University of Amsterdam	2	1.527
6	Austrian Science Fund Fwf	2	1.527
7	Deutsche Arthrose Hilfe E V	2	1.527
8	Projekt Deal	2	1.527
7	Swiss National Science Foundation Snsf	2	1.527
10	Amniox Medical	1	0.763

### 3.4 Analysis of journal co-citations

**Figure 5:**
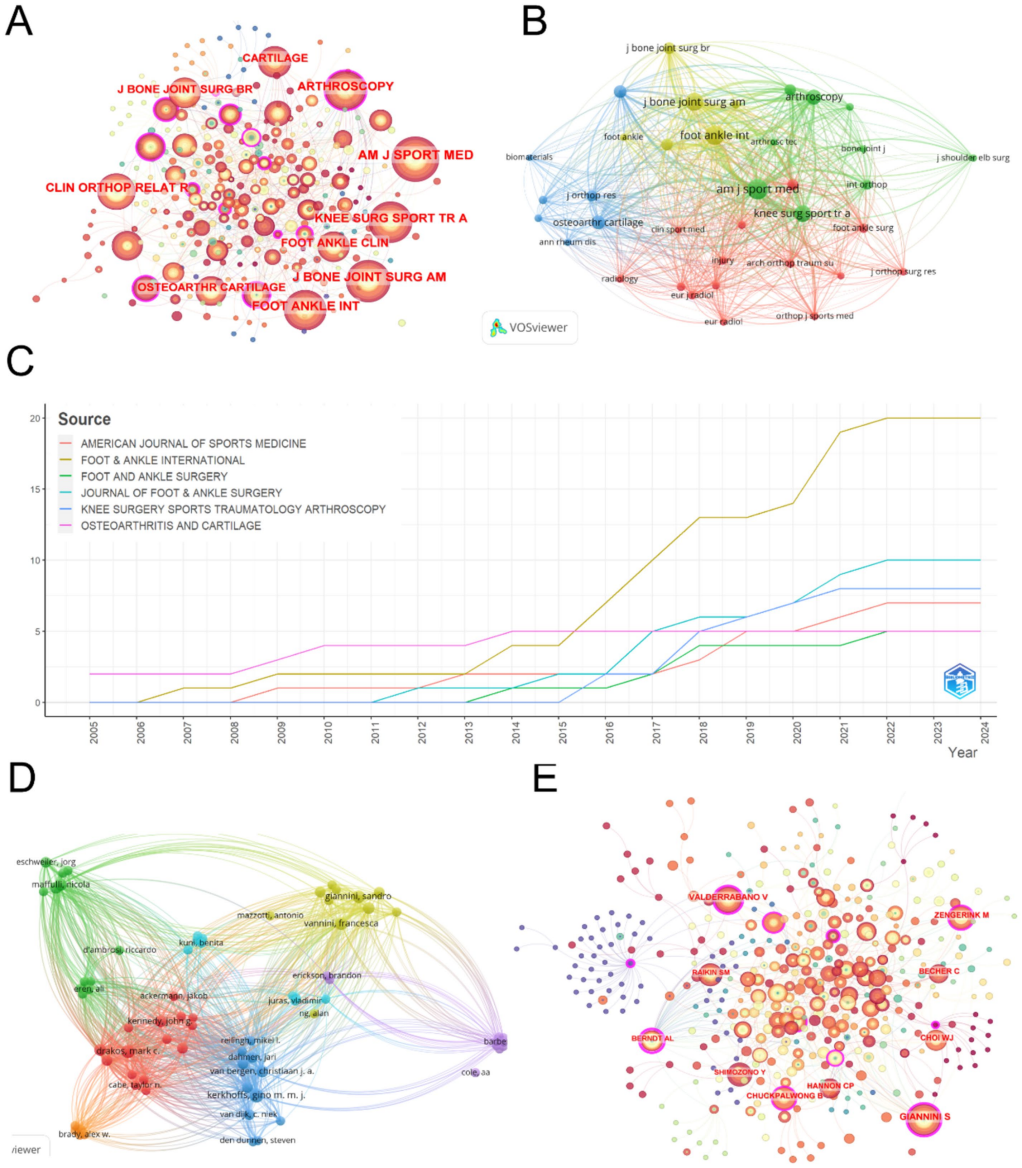
(A) Journal co-citation analysis on CiteSpace. (B) Co-citation analysis of journals with more than 20 citations based on Vosviewer. (C) Change in total number of publications in the top 6 most productive journals from 2004 to 2024. (D) Collaboration analysis of authors with more than 2 publications based on VOSviewer. (E) Author collaboration analysis on CiteSpace.

Figure 5A presents the co-citation analysis of journals in the field of ankle cartilage repair. In this analysis, concentric circle nodes represent journals, with the color and thickness of the rings reflecting the year and number of citations, respectively. The connecting lines between nodes represent co-citations, with the thickness of the lines indicating the frequency of these co-citations. Nodes highlighted in purple indicate journals with high centrality in the co-citation network, signifying their influential role in connecting different areas of research.

The top five most cited journals identified through this analysis are: (1) *American Journal of Sports Medicine* (AM J SPORT MED) with 642 citations and a total link strength of 20,084. (2) *Foot and Ankle International* (FOOT ANKLE INT) with 535 citations and a total link strength of 16,425. (3) *Journal of Bone and Joint Surgery-American Volume* (J BONE JOINT SURG AM) with 406 citations and a total link strength of 12,319. (4) *Knee Surgery Sports Traumatology Arthroscopy* (KNEE SURG SPORT TR A) with 350 citations and a total link strength of 12,049. (5) *Arthroscopy: The Journal of Arthroscopic and Related Surgery* (ARTHROSCOPY) with 246 citations and a total link strength of 8,461. Notably, except for *Foot & Ankle International*, which is ranked in the second quartile (Q2) of the Journal Citation Reports (JCR), the other journals are ranked in the first quartile (Q1), indicating their high impact and prominence in the field. Additionally, *Arthroscopy*, *Osteoarthritis and Cartilage*, and *Foot and Ankle International* exhibit high centrality within the co-citation network, highlighting their significant role in shaping research and serving as pivotal sources of information within the domain of ankle cartilage repair.

Building on the analysis in Figure 5A, we filtered out 36 journals that were cited more than 20 times and used VOSviewer to create Figure 5B. In this visualization, the node size represents the total number of citations, while the lines between nodes carry the same meaning as before, indicating co-citation relationships. Journals of the same color form clusters, which suggest that journals within the same cluster are co-cited more frequently, reflecting similar research directions.

Among the four identified clusters: (1) The red, yellow, and green clusters include journals primarily related to the treatment of ankle cartilage injuries. These clusters focus on the application of current treatments and are frequently cited for reviewing existing therapies and providing theoretical and empirical support for these methods. (2) The blue cluster encompasses journals that cover a broader range of disciplines, including life sciences, engineering, translational research, and clinical studies on new advancements in ankle cartilage repair technologies. These journals are mainly cited for offering technical support and innovation in research, indicating their role in pioneering new techniques and methodologies in the field.

Table 4 lists the top 10 most productive journals in the field of ankle cartilage repair. *Foot and Ankle International* ranks first with 20 articles, followed by the *Journal of Foot and Ankle Surgery* with 10 articles, and *Knee Surgery Sports Traumatology Arthroscopy* with 8 articles. Figure 5C illustrates the cumulative number of articles published from 2004 to 2024 by the top six high-production journals. From 2004 to 2015, the publication rate of these journals remained relatively stable. However, starting in 2016, *Foot and Ankle International* significantly accelerated its publication rate, surpassing other journals and establishing itself as a leading platform for research in the field of ankle cartilage repair.

**Table 4:** The top 10 most productive journals related to ankle cartilage repair from 2004 to 2024.

Rank	Journal	Article counts	IF
1	*Foot and Ankle International*	20	2.4
2	*Journal of Foot Ankle Surgery*	10	1.3
3	*Knee Surgery Sports Traumatology Arthroscopy*	8	3.3
4	*American Journal of Sports Medicine*	7	4.2
5	*Foot and Ankle Surgery*	5	1.9
6	*Osteoarthritis and Cartilage*	5	7.2
7	*Cartilage*	4	2.7
8	*Clinical Orthopaedics And Related Research*	3	4.2
9	*Journal of Orthopaedic Surgery and Research*	3	2.8
10	*Operative Techniques in Sports Medicine*	3	0.4

### 3.5 Analysis of author collaborations

Table 5 lists the top 10 most productive authors in this field. Kerkhoffs GMMJ leads with 11 articles, followed by Vannini F with 9 articles. Buda R, Giannini S, and Van Dijk CN are tied for third place, each with 6 articles.

**Table 5:** The top 10 authors with the most publications on ankle cartilage repair from 2004 to 2024.

Rank	Highly Published Authors	Article counts	Percentage (%)
1	Kerkhoffs GMMJ	11	8.397
2	Vannini F	9	6.87
3	Buda R	7	5.344
4	Giannini S	7	5.344
5	Van Dijk CN	7	5.344
6	Dahmen J	6	4.58
7	Drakos MC	6	4.58
8	Kennedy JG	6	4.58
9	Stufkens SAS	6	4.58
10	Van Bergen CJA	6	4.58

Analyzing the authorship of these publications helps identify the key contributors and core research groups in this field. The renowned scholar Price (71) observed that half of the papers on a given subject are typically authored by a small group of high-productivity researchers. According to Price’s Law, the number of these prolific authors is approximately equal to the square root of the total number of contributors in the field.


$$\overset{I}{\underset{m+1}{\sum }}n\left(x\right)=\sqrt{N}$$

The variable $n\left(x\right)$ represents the number of authors who have written x papers, and $I=n_{\text{max}}$ represents the highest number of papers by a single author in this field. According to VOSviewer, $n_{\text{max}}$ is 11 papers. Let N be the total number of authors, and m be the minimum number of papers published by core authors. According to Price’s Law, the minimum number of papers for core authors in the field of ankle cartilage repair is calculated as m = 0.749× $\sqrt{n_{\text{max}}}$ ≈2.48. Therefore, authors with at least 2 papers are considered core authors, theoretically amounting to 73 core authors. However, the top 10 most prolific authors listed in Table 5 alone have published 71 papers, accounting for 54.2% of the total publications. This does not align with Price’s Law’s half-standard, suggesting that in the field of ankle cartilage repair, a very small number of core authors have published the majority of the papers. In other words, it indicates that the field has not yet formed a relatively stable research community, with research activities primarily driven by a few key researchers. Figure 5E, generated using CiteSpace, depicts the author collaboration network. Concentric circle nodes represent authors, with the color and thickness of the rings indicating the year and citation frequency, respectively. The lines represent collaboration, with the thickness of the lines indicating the frequency of collaboration. Nodes highlighted in purple have high centrality in the collaboration network. Authors with higher citation counts include Giannini S (377 citations, total link strength = 2088), Vannini F (364 citations, total link strength = 2,631), Buda R (356 citations, total link strength = 2,213), and Kerkhoffs GMMJ (205 citations, total link strength = 3,798). In the author collaboration network, Giannini S, Valderrabano V, and Zengerink M show high centrality. Based on Figure 5E, we filtered out 73 authors with more than 2 publications and used VOSviewer to create Figure 5D. In this figure, the node size represents the total number of citations, with the same line meanings as before. Authors of the same color form a cluster, indicating that they collaborate more frequently and have similar research directions.

### 3.6 Analysis of references

Table 6 lists the top 10 most cited research papers and reviews in the field of ankle cartilage repair. The most cited paper is a research article titled “One-step Bone Marrow-derived Cell Transplantation in Talar Osteochondral Lesions,” published in 2009 in *Clinical Orthopaedics and Related Research*, with 198 citations. The second most cited is a review titled “Cartilage degeneration in different human joints,” published in 2004 in *Osteoarthritis and Cartilage*, with 152 citations. The third most cited paper is a research article titled “One-Step Repair in Talar Osteochondral Lesions: 4-Year Clinical Results and T2-Mapping Capability in Outcome Prediction,” published in 2013 in the *American Journal of Sports Medicine*, with 124 citations.

**Table 6:** The top 10 research/review articles with the most citations in the field of ankle cartilage repair from 2004 to 2024.

Rank	Title	Article/review	Journal	Publication year	Total citations
1	One-step Bone Marrow-derived Cell Transplantation in Talar Osteochondral Lesions	Article	*Clinical Orthopaedics and Related Research*	2009	198
2	Cartilage degeneration in different human joints	Review	*Osteoarthritis and Cartilage*	2004	152
3	One-Step Repair in Talar Osteochondral Lesions 4-Year Clinical Results and T2-Mapping Capability in Outcome Prediction	Article	*American Journal of Sports Medicine*	2013	124
4	Arthroscopic Treatment of Osteochondral Defects of the Talus Outcomes at Eight to Twenty Years of Follow-up	Article	*Journal of Bone and Joint Surgery-American Volume*	2013	116
5	*In vivo* cartilage contact strains in patients with lateral ankle instability	Article	*Journal of Biomechanics*	2024	87
6	Stem cells in articular cartilage regeneration	Review	*Journal of Orthopaedic Surgery and Research*	2016	84
7	The detached osteochondral fragment as a source of cells for autologous chondrocyte implantation (ACI) in the ankle joint	Article	*Osteoarthritis and Cartilage*	2004	72
8	Autologous Chondrocyte Implantation of the Ankle A 2-to 5-Year Follow-Up	Article	*American Journal of Sports Medicine*	2009	71
9	Matrix-Induced Autologous Chondrocyte Implantation (MACI) Grafting for Osteochondral Lesions of the Talus	Article	*Foot and Ankle International*	2009	60
10	Comparison of Osteochondral Autografts and Allografts for Treatment of Recurrent or Large Talar Osteochondral Lesions	Article	*Foot and Ankle International*	2016	55

**Figure 6:**
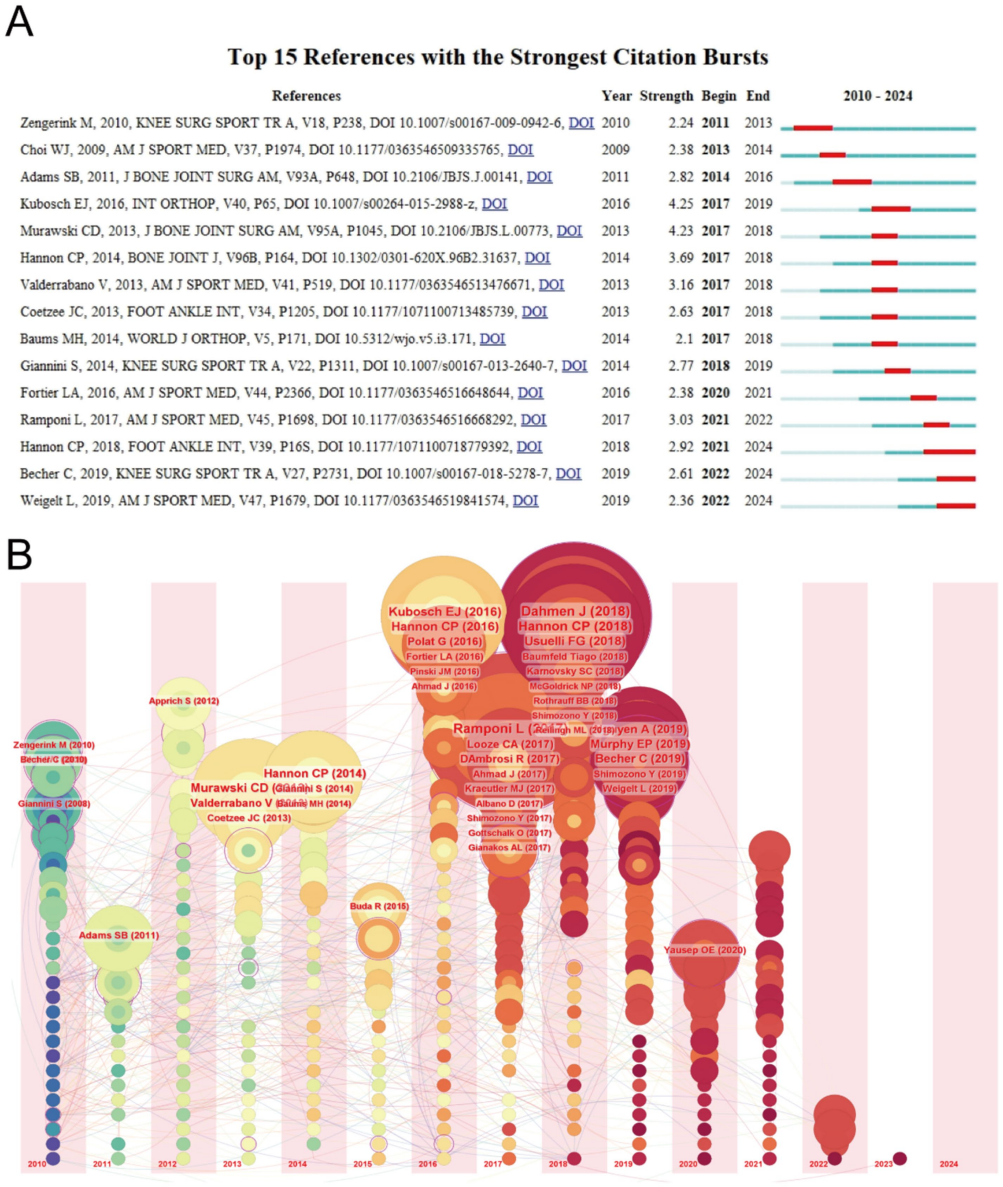
(A) Top 15 references with the strongest citation bursts of publications related to ankle cartilage repair from 2010 to 2024. (B) Timeline visualization of references in ankle cartilage repair from 2010 to 2024.

Using CiteSpace, we identified the top 15 papers with the highest citation burst strength in the field of ankle cartilage repair from 2010 to 2024 (Figure 6A). The paper with the highest citation burst strength (4.25) is titled “Clinical outcome and T2 assessment following autologous matrix-induced chondrogenesis in osteochondral lesions of the talus,” authored by Kubosch EJ et al. and published in 2016 in *International Orthopaedics*. The second highest citation burst strength (4.23) belongs to the paper titled “Operative treatment of primary anterior cruciate ligament rupture in adults,” authored by Murawski CD et al. and published in 2013 in the *Journal of Bone and Joint Surgery-American Volume*. These two papers have citation burst strengths that significantly exceed those of others. Additionally, the paper titled “Debridement, Curettage, and Bone Marrow Stimulation: Proceedings of the International Consensus Meeting on Cartilage Repair of the Ankle,” authored by Hannon CP et al. and published in 2018 in *Foot and Ankle International*, has the longest citation burst duration, lasting from 2021 to 2024.

Figure 6B provides a timeline visualization of references, where concentric circle nodes represent references, with the color and thickness of the rings indicating the year and citation frequency, respectively. The horizontal axis represents the publication year of the references, and the lines depict the citation relationships between them. It is evident that many highly cited papers were published between 2016 and 2018, consistent with the earlier observation that the field of ankle cartilage repair has seen rapid development since 2016.

### 3.7 Analysis of keywords

**Figure 7:**
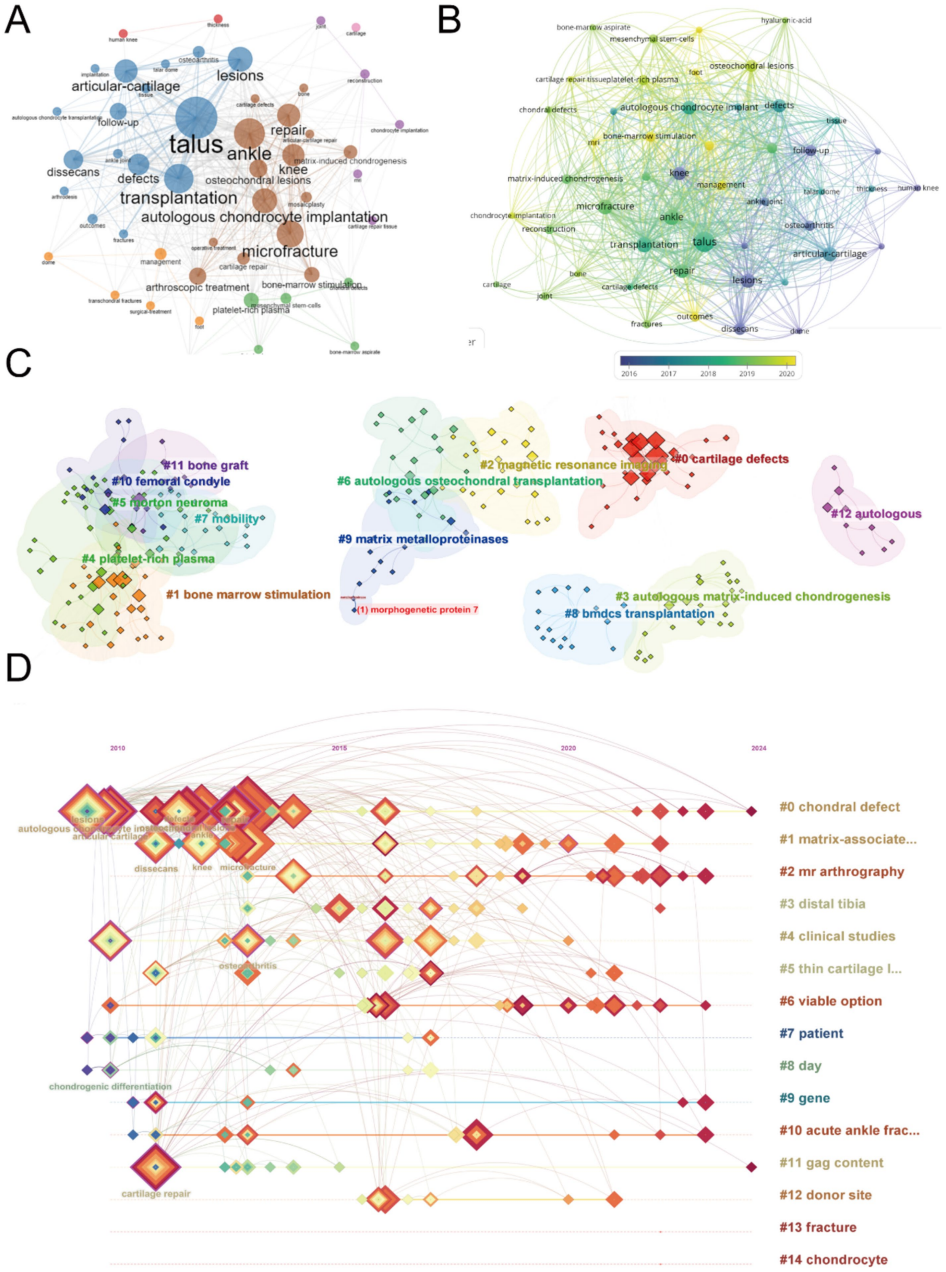
Mapping of keywords in studies on ankle cartilage repair. (A) Co-occurrence analysis of keywords based on VOSviewer. (B) Distribution of keywords according to average publication year (blue: earlier, yellow: later) by VOSviewer. (C) Clustering analysis of the keyword network based on CiteSpace. (D) Keyword timeline visualization from 2010 to 2024 by CiteSpace.

A co-occurrence analysis of keywords was conducted to identify research hotspots and directions in the field of ankle cartilage repair. A total of 49 keywords appeared more than five times, and these were visualized using VOSviewer (Figure 7A). In this network visualization, the node size represents the frequency of keyword appearances, while the lines represent keyword co-occurrences. Keywords of the same color form clusters, where those within the same cluster co-occur more frequently, indicating similar research directions. The most frequently appearing keywords include “talus” (300 occurrences), “ankle” (280), “transplantation” (270), and “microfracture” (230), among others.

To further explore the trends, we visualized the average publication year of these keywords (Figure 7B). In this figure, the node color transitions from dark to light, representing the average publication year from earlier to more recent times. The analysis reveals that high-frequency keywords primarily appeared between 2017 and 2019. Newer and influential keywords that have emerged include “bone-marrow stimulation,” “osteochondral lesions,” “mesenchymal stem-cells,” and “chondrocyte implantation.”

Next, we clustered and numbered the keywords using CiteSpace (Figure 7C), dividing them into 15 keyword clusters, labeled from #0 to #14. The timeline of each keyword cluster was then visualized (Figure 7D). In this timeline, concentric square nodes represent keywords, with the color and thickness of the square rings indicating the year and frequency of keyword occurrences, respectively. The horizontal axis represents the first year a keyword appeared, and the lines show co-occurrences between keywords. Keywords highlighted in purple are particularly impactful. The analysis shows that while hotspot keywords such as “lesion,” “articular cartilage,” “repair,” “osteoarthritis,” and “autologous chondrocyte transplantation” mainly appeared between 2010 and 2015, they have remained active beyond 2020. These hotspot keywords predominantly belong to clusters #0 (chondral defect) and #1 (matrix-associated).

**Figure 8:**
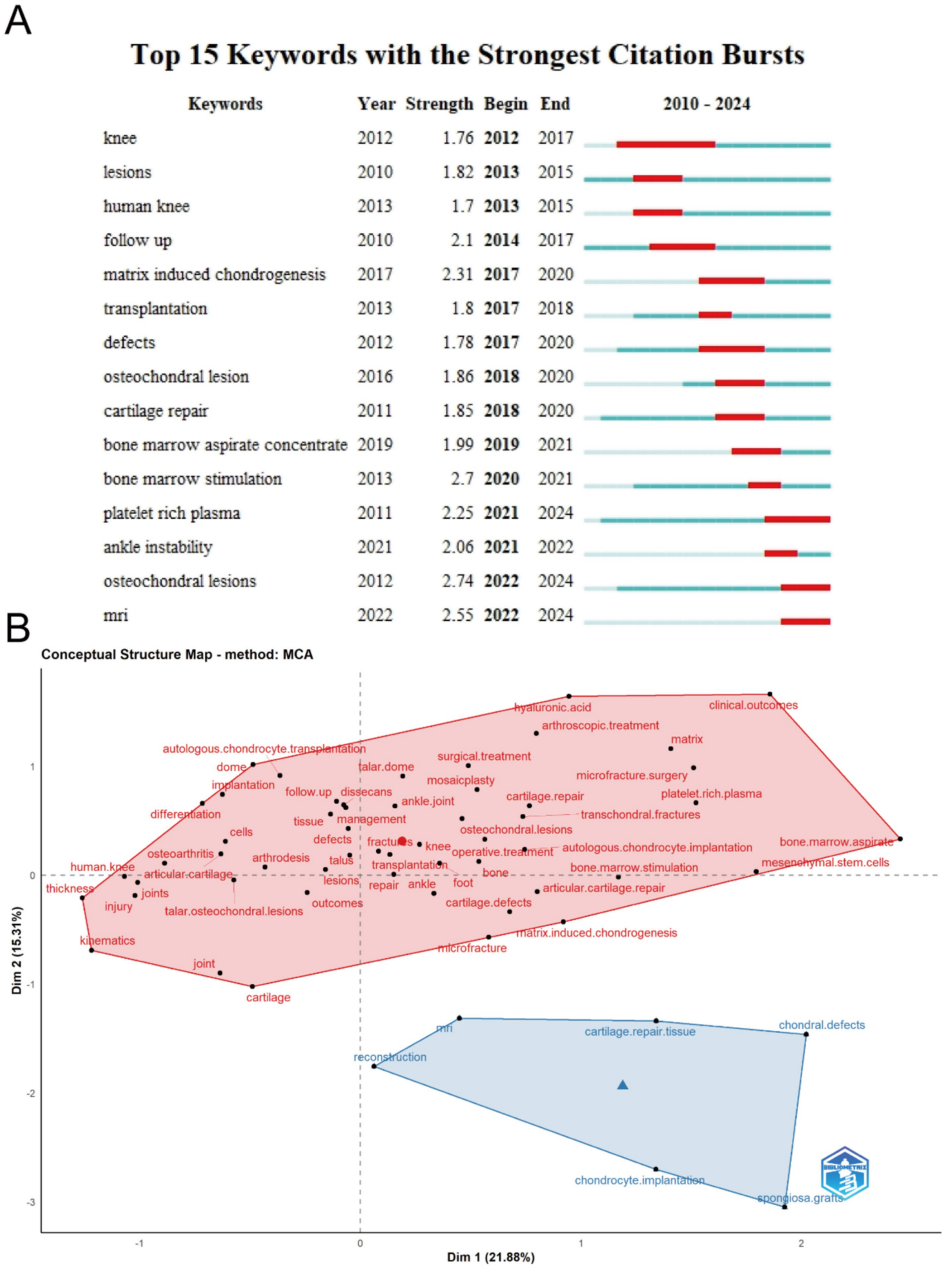
(A) Top 15 keywords with the strongest citation bursts of publications related to ankle cartilage repair from 2010 to 2024. (B) Conceptual structure map of keywords in studies on ankle cartilage repair by Multiple Correspondence Analysis.

Figure 8A highlights the top 15 keywords with the highest citation burst strength in the field of ankle cartilage repair from 2004 to 2024. The keyword “osteochondral lesions” (burst strength of 2.74), which first appeared in 2012, has the highest citation burst strength. “MRI” (burst strength of 2.55), appearing in 2022, ranks second, and “matrix-induced chondrogenesis” (burst strength of 2.31), appearing in 2017, ranks third. The keyword “knee,” which appeared in 2012, has the longest citation burst duration, lasting from 2012 to 2017. Keywords such as “osteochondral lesions” and “platelet-rich plasma,” which appeared early, have experienced citation bursts in recent years, indicating significant advancements and continued relevance, warranting attention.

Figure 8B presents a concept structure map generated through Multiple Correspondence Analysis (MCA), where the characteristics of keywords are simplified into horizontal and vertical coordinate values. Keywords in the field of ankle cartilage repair are grouped into two categories, blue and red. The red cluster encompasses keywords related to descriptions of ankle cartilage injuries and existing clinical therapies and surgical techniques. The blue cluster includes keywords associated with experimental repair methods, imaging techniques like “MRI,” and terms related to tissue engineering, such as “reconstruction,” “chondrocyte implantation,” “cartilage repair tissue,” and “spongiosa graft.”

## 4 Discussion

### 4.1 Research status

Our team conducted a bibliometric analysis of papers on ankle cartilage repair published between 2004 and 2024 to explore the progress and future directions in this field. The year 2016 marked a significant uptick in publications and research interest, with *Foot and Ankle International* emerging as a key journal. We anticipate that this trend will continue through 2026. The United States leads in several metrics, including publications, citations, and H-index, with five of the top 10 contributing institutions and four of the top 10 funding sources. However, collaboration is predominantly within countries, suggesting a need for increased international cooperation. To enhance this cooperation, researchers could create international research consortia focused on ankle cartilage injuries. These networks can facilitate shared knowledge, resources, and data, allowing researchers to work together on large-scale studies and clinical trials. We can also use platforms like the Global Burden of Disease study to identify hotspots for ankle injuries, directing collaborative efforts to regions with high incidence rates. The international cooperation also needs the researchers to develop universally accepted guidelines for the diagnosis, treatment, and rehabilitation of ankle cartilage injuries. This can include standard imaging techniques, surgical approaches, and post-operative care.

Data from the Journal Citation Reports (JCR) website indicate that among the top 10 journals in this field, only *Osteoarthritis and Cartilage* has an impact factor above 5 (IF 7.2). The impact factors of other leading journals range from 2 to 4, reflecting the specialized nature of this research area. Exceptions include *Cartilage* and the *Journal of Orthopaedic Surgery and Research*, which are open access journals that expedite the review and dissemination process. *Foot and Ankle International*, a subscription-based journal founded in 1980, has published 20 articles (15% of all articles in our dataset) and continues to be highly influential. We recommend considering this journal for submissions, as well as open access options for faster publication.

The leading research areas in ankle cartilage repair are Orthopedics, Surgery, and Sports Sciences, with notable interdisciplinary contributions from Engineering, Cell Biology, Biotechnology, Applied Microbiology, and Biophysics. Advanced therapies involving stem cells and tissue engineering scaffolds are under active development. The most influential author identified in our analysis is Kerkhoffs GMMJ from the Netherlands, known for his work in arthroscopic surgery and ankle injuries. Other notable scholars include Giannini S and Vannini F from Italy, and Valderrabano V from Switzerland.

A co-citation analysis highlighted that the most cited paper in this field is “One-step Bone Marrow-derived Cell Transplantation in Talar Osteochondral Lesions,” which has been instrumental in guiding significant advancements. Of the top 10 most cited papers, 8 are research articles and 2 are reviews, underscoring the need for more comprehensive reviews to synthesize the growing body of knowledge in ankle cartilage repair.

### 4.2 Research hotspots and emerging directions

The co-occurrence analysis of keywords offers a deeper insight into the research priorities within the field of ankle cartilage repair, while citation bursts of individual keywords highlight emerging research hotspots. We have organized these hotspots into three main categories: risk factors and pathogenesis, current clinical treatments, and advanced regenerative technologies.

#### 4.2.1 Risk factors and pathogenesis

Understanding the risk factors and pathogenesis of ankle cartilage injury is fundamental to addressing and preventing these injuries. Keyword analysis reveals that “trauma,” “sports injuries,” and “degenerative changes” are frequently cited as primary causes. Compared to the knee joint, the ankle cartilage has a higher extracellular matrix density, with increased proteoglycan and water content, resulting in greater stiffness and lower permeability (39). This structure endows ankle cartilage with a stronger load-bearing capacity but reduces its sensitivity to mechanical injury. Research indicates that ankle trauma, including sprains and fractures, as well as repetitive movements—especially those involving jumping and running—are major contributors to cartilage damage and degeneration (40, 41). Consequently, 70 to 78% of ankle osteoarthritis (OA) cases are categorized as posttraumatic osteoarthritis (PTOA) (42). Additionally, factors such as obesity, aging, and congenital genetic factors (including joint laxity, foot anatomical morphology, and leg muscle imbalance) are also implicated in ankle cartilage degeneration (6). In a study by Garrido C P et al., *ex vivo* simulation of ankle cartilage injury was conducted using a 4 mm cylindrical indenter, applying a single impact with an impulse of 1 Ns to the talus of a healthy individual (43). The impact resulted in the immediate death of more than two-thirds of the cartilage cells in the directly damaged area, and within 7 days, apoptosis and cartilage degeneration had spread radially to surrounding regions (43). T Trauma often initiates a cascade of events in ankle cartilage that ultimately leads to osteoarthritis through three main pathways: Direct cartilage rupture with subchondral bone injury due to trauma. Cartilage cell apoptosis indirectly caused by trauma, such as intra-articular fractures. Long-term post-traumatic biomechanical issues, including malalignment, malunion after fractures, or ligament laxity, which lead to extensive superficial or erosive cartilage damage (44). The gradual degeneration of ankle cartilage is a highly complex process involving mechanical, inflammatory, and metabolic factors (45). An imbalance between joint tissue repair and destruction ultimately leads to overall joint damage and dysfunction, with matrix metalloproteinase activation and the release of pro-inflammatory growth factors playing crucial roles in this process (46).

#### 4.2.2 Current clinical treatments

Current clinical treatments are a major focus in ankle cartilage repair research. Keyword analysis frequently highlights “microfracture,” “autologous cartilage transplantation,” and “allogeneic cartilage transplantation,” all of which are widely applied and studied in clinical settings.

Microfracture (MF) is a technique that stimulates cartilage regeneration by creating small holes in the cartilage defect area, promoting the release of bone marrow stem cells and growth factors. Due to its minimal invasiveness and low postoperative complication rate, microfracture is currently a frontline treatment for smaller osteochondral lesions (47). However, the regenerated fibrocartilage produced through this method has inferior mechanical strength compared to hyaline cartilage and tends to degenerate over time (48). To enhance the repair effect, biologic compounds are often used during and after surgery, which can improve clinical outcomes for cartilage injuries, such as osteoarthritis. Commonly studied compounds include hyaluronic acid (HA), known for its lubricating properties, and platelet-rich plasma (PRP), which contains high concentrations of platelets and growth factors (49, 50). A prospective randomized clinical trial demonstrated that PRP and HA injections, as adjunctive therapies, improved outcomes of arthroscopic microfracture in treating talar osteochondral lesions (OCL) over a moderate follow-up period (15.3 months). A single dose of PRP was recommended as the primary adjuvant therapy for postoperative talar OCL (51).

Autologous and allogeneic cartilage transplantation techniques involve transplanting healthy cartilage tissue to repair defect areas. Despite over two decades of clinical use, several challenges remain. Autologous cartilage transplantation is limited for large defects due to insufficient cartilage cell sources, lengthy cartilage cell harvesting processes, fixation difficulties, periosteal hypertrophy, and ablation, as well as reduced efficacy in elderly patients (38). Moreover, autologous chondrocyte implantation cannot effectively repair the osteochondral interface and full-thickness cartilage, often causing damage to the subchondral bone at the donor site. Allografts face challenges such as limited tissue supply, immune rejection, poor integration, decreased cell viability due to graft storage, and potential disease transmission (52). To improve treatment outcomes and minimize side effects, researchers are exploring new transplantation materials. Degradable polymer scaffolds combined with cartilage cells have shown promising results in cartilage regeneration, effectively circumventing the drawbacks of requiring two surgeries and the high costs associated with autologous chondrocyte implantation. For instance, Giannini S et al. developed a scaffold using collagen powder or a hyaluronic acid membrane combined with platelet gel, which is loaded with concentrated bone marrow-derived stem cells (BMSCs) for one-step arthroscopic transplantation to treat talar osteochondral defects (53). This approach has shown potential in enhancing cartilage repair while reducing the complexities associated with traditional transplantation methods.

#### 4.2.3 Frontiers in regenerative technologies

Frontier regenerative technologies represent the latest advancements in ankle cartilage repair, with keywords such as “stem cell therapy, ““tissue engineering,” and “gene therapy” appearing with notable frequency.

##### 4.2.3.1 Stem cell therapy

Mesenchymal stem cells (MSCs) are considered one of the most promising therapies for osteoarthritis due to their ability to proliferate in an undifferentiated, multipotent state (self-renewal) and their capacity to differentiate into multiple tissue-specific cell types (54). Stem cell therapy has shown encouraging results in both animal models and early clinical trials. Currently, MSCs used for cartilage tissue engineering are primarily sourced from bone marrow (BMSCs), though they can also be derived from other tissues such as adipose tissue (adipose-derived stem cells - ADSCs), amniotic fluid (AFSCs), synovium, and periosteum (55). In a long-term follow-up study, Emadedin et al. (56) observed that patients with ankle osteoarthritis who received BMSC injections experienced no severe adverse reactions over 30 months and showed effective therapeutic results. Additionally, induced pluripotent stem cells (iPSCs) and stem cell-derived exosomes are emerging as superior alternatives for stem cell therapy, as they circumvent ethical concerns associated with other stem cell sources (55). Zhang et al. (57) were the first to discover that intra-articular injection of exosomes derived from human embryonic mesenchymal stem cells could promote the regeneration of osteochondral defects in rats. Compared to direct exosome injection, designing auxiliary delivery systems can help exosomes remain at the defect site for an extended period and be released gradually, thereby reducing the discomfort associated with repeated injections (58). For example, Liu et al. (59) developed an acellular tissue patch (EHG) composed of exosomes derived from hiPSC-MSCs, encapsulated in a photoinduced imine crosslinking hydrogel glue. The EHG tissue patch can integrate with the native cartilage matrix, representing a novel acellular repair approach. These advancements in stem cell therapy, including the development of exosome-based treatments and innovative delivery systems, highlight the potential of regenerative technologies to revolutionize ankle cartilage repair.

##### 4.2.3.2 Tissue engineering

Cartilage tissue engineering typically involves embedding chondrocytes or stem cells in supportive matrices, such as hydrogels or scaffolds, to induce differentiation along the cartilage lineage (60). In recent years, advancements in nanotechnology and 3D printing have continuously driven innovation in scaffold construction. These new scaffolds more closely mimic the extracellular matrix (ECM) microenvironment, enhancing chondrocyte adhesion, proliferation, and differentiation. Additionally, they offer superior mechanical properties and biocompatibility, leading to significantly improved cartilage repair outcomes. As a result, these advanced scaffolds are gradually being translated into clinical applications for joint cartilage repair (61, 62). For instance, Christensen et al. (63) conducted a follow-up study on patients with knee and ankle cartilage lesions treated with the MaioRegen^®^ scaffold—a cell-free biomimetic scaffold composed of type I collagen and hydroxyapatite—over a period of 1–3 years. The study observed improved clinical outcomes, demonstrating the scaffold’s potential in cartilage repair (63). Similarly, Di Cave et al. (64) performed a retrospective study that highlighted the effectiveness of a biphasic bioresorbable scaffold designed to stimulate both cartilage and subchondral bone regeneration in treating osteochondral lesions of the talus. These studies underscore the promising role of advanced scaffolds in enhancing the repair and regeneration of cartilage tissue, marking significant progress in the clinical translation of cartilage tissue engineering technologies.

##### 4.2.3.3 Gene therapy

With advancements in gene delivery systems, gene therapy has emerged as a promising potential treatment for cartilage disorders. Unlike traditional treatments that involve short-lived molecules, gene therapy can introduce therapeutic genes, potentially extending the duration of therapeutic effects—an especially valuable benefit for progressive diseases. Combining gene therapy with stem cell and tissue engineering strategies to create an integrated treatment approach represents a feasible and innovative research direction that could maximize cartilage repair outcomes. Identified candidate genes for this approach include transforming growth factor beta (TGF-*β*), bone morphogenetic proteins (BMPs), basic fibroblast growth factor (FGF-2), insulin-like growth factor I (IGF-I), and various transcription factors that regulate cell differentiation, such as the Sox and Ets families (65). Current methods for delivering gene sequences to cartilage injury sites include: (1) Injection of the therapeutic composition (gene vector, genetically modified differentiated or progenitor cells) intra-articularly inside the joint space. (2) Administration of the therapeutic composition via open joint surgery (arthrotomy) (65). Cucchiarini M et al. conducted a series of experiments using recombinant adeno-associated virus (rAAV) as a vector to deliver the gene sequences of fibroblast growth factor 2 (FGF-2) (66), IGF-1 (67), and the transcription factor SOX9 (68) to the osteochondral defect sites in rabbits. The therapeutic vector facilitated significant repair effects by promoting the overexpression of the target genes. Additionally, Murphy et al. (69) implanted retrovirally modified MSCs into a goat osteoarthritis (OA) model, resulting in reduced cartilage damage. Hu et al. (70) injected Bcl-xL engineered mesenchymal stem cells into a rabbit OA model, enhancing MSC implantation and therapeutic efficacy. These promising results in large animal models suggest that the clinical translation of gene therapy for ankle cartilage repair holds great potential for the future, offering hope for more effective and sustained treatment options for cartilage disorders.

### 4.3 Future research directions

Based on the results of this bibliometric analysis, we propose the following suggestions for the future development of the field of ankle cartilage repair: **(1) In-Depth Exploration of the Molecular Mechanisms of Cartilage Injury:** Research on the molecular mechanisms underlying cartilage injury is still limited. Future studies should focus on investigating the molecular processes of chondrocytes during injury and repair using genomics, proteomics, and metabolomics. This approach will provide a theoretical foundation for developing new treatment strategies. Advances in high-throughput sequencing and mass spectrometry now allow for a more comprehensive understanding of genetic, proteomic, and metabolic changes in chondrocytes, helping to identify potential therapeutic targets and deepen the understanding of injury mechanisms. **(2) Optimization of Existing Clinical Treatment Methods:** Current clinical treatments have significant limitations. For example, cartilage regenerated after microfracture often has poor quality and is prone to degeneration. Autologous and allogeneic cartilage transplantation face challenges such as limited donor availability and immune rejection. To improve treatment outcomes and patient prognosis, integrating advanced biomaterials and technologies is essential. For instance, further development of adjuvant biological compounds based on PRP and HA could enhance clinical outcomes. Personalized cartilage scaffolds created using 3D printing technology, which incorporate drug delivery systems or reactive microenvironment control systems, offer a promising avenue. Additionally, nanotechnology-based biomaterials could provide superior mechanical properties and biocompatibility, improving the function and durability of transplanted cartilage. **(3) Promoting Clinical Applications of Stem Cell and Gene Therapy:** Large-scale clinical trials are crucial for verifying the safety and efficacy of stem cell and gene therapies in cartilage repair, paving the way for their clinical application. Alternatives such as iPSCs or stem cell-derived exosomes could be considered to avoid ethical concerns. These therapies can be combined with tissue engineering scaffolds to further enhance cartilage repair outcomes, providing a more effective and integrated approach to treatment. Generally, we should advocate for research that combines genetic, biomechanical, and material science data to develop personalized treatment plans for ankle cartilage injuries. Interdisciplinary collaboration can help tailor interventions based on individual patient needs and injury profiles. **(4) Interdisciplinary Collaboration and Precision Medicine:** The future of cartilage repair research lies in interdisciplinary collaboration, particularly with fields such as biomedical science, materials science, and engineering. We should encourage interdisciplinary teams to explore cutting-edge technologies such as 3D bioprinting and regenerative medicine techniques. These innovations can lead to breakthroughs in how cartilage is repaired or replaced. For 3D printing technologies, which can facilitate the customization of scaffolds that mimic the mechanical and biological properties of native cartilage, potentially enhancing repair outcomes. Moreover, advances in bioengineering can lead to the development of bioactive scaffolds that promote cellular adhesion and proliferation, further improving graft integration and function. Additionally, incorporating data analytics and computational modeling from computer science can enhance treatment strategies by leveraging machine learning to analyze patient-specific data. This approach can help identify optimal candidates for specific therapies, improving patient selection and resource allocation. Future research should also focus on precision medicine by developing personalized treatment plans tailored to the patient’s clinical presentation and lifestyle. Long-term follow-up studies are essential to optimize treatment plans, minimize unnecessary interventions, and reduce side effects. Additionally, epidemiological analyses of specific cases can enrich clinical experience, gradually leading to more refined, personalized treatment pathways. **(5) Prevention and Early Intervention:** With osteoarthritis now the fourth leading cause of disability worldwide, prevention and early intervention are crucial. Future research should focus on high-risk groups prone to ankle cartilage damage, such as fitness enthusiasts and athletes. Through health education, exercise guidance, and regular screening, the incidence of cartilage injuries can be reduced. Furthermore, the development of new early diagnostic and intervention techniques, such as biomarker detection and high-resolution imaging, will be a promising research direction. By addressing these areas, future research in ankle cartilage repair can lead to significant advancements, improving both preventive and therapeutic strategies and ultimately enhancing patient outcomes.

### 4.4 Strengths and limitations

Despite utilizing bibliometric methods to comprehensively and objectively analyze the literature on ankle cartilage repair over the past 20 years, our study has certain limitations. First, our selection of publications was restricted to the Web of Science Core Collection (WOSCC), excluding other databases such as PubMed, Scopus, Cochrane, and Embase. While WOSCC is widely regarded as a comprehensive and rich database, relevant literature from other sources may have been omitted, potentially introducing selection bias. Secondly, our analysis was limited to English-language publications, which excluded a significant number of non-English studies. This is particularly noteworthy given the important contributions from Italian and Dutch researchers in this field. Lastly, recent high-quality publications may not yet have accumulated sufficient citations to be prominently featured in our bibliometric analysis, which could lead to discrepancies between our findings and the current state of the field. Therefore, we recommend that researchers stay updated with the latest publications, including those in non-English languages, to ensure a comprehensive and current understanding of the field of ankle cartilage repair.

## 5 Conclusion

This study illustrates the dynamic landscape of ankle cartilage repair from 2004 to 2024, highlighting the steady increase in global attention driven by advancements in tissue engineering and regenerative medicine. The United States, with its leading academic institutions, has produced the highest number of high-quality articles, underscoring its leadership in the field. Through keyword and cluster analysis, we identified key research hotspots and future directions: in-depth exploration of the molecular mechanisms underlying cartilage injury to develop innovative therapeutic strategies; optimization of existing clinical treatment methods to improve patient outcomes, reduce long-term complications, and minimize the need for invasive surgery; and the advancement of clinical translation of stem cell and gene therapies, which could revolutionize the treatment landscape. Additionally, strengthening interdisciplinary collaboration to promote precision medicine, with a focus on prevention and early intervention, is essential. We hope this research will guide scholars toward promising development directions, provide valuable references for policymakers and administrators, and ultimately promote the continuous advancement of the field.

## Data Availability

The original contributions presented in the study are included in the article/supplementary material, further inquiries can be directed to the corresponding authors.
